# Knock-in rats with homozygous *PSEN1^L435F^* Alzheimer mutation are viable and show selective γ-secretase activity loss causing low Aβ40/42 and high Aβ43

**DOI:** 10.1074/jbc.RA120.012542

**Published:** 2020-04-07

**Authors:** Marc D. Tambini, Luciano D'Adamio

**Affiliations:** Department of Pharmacology, Physiology, and Neuroscience, the Brain Health Institute, the Jacqueline Krieger Klein Center in Alzheimer's Disease and Neurodegeneration Research of Rutgers New Jersey Medical School, Newark, New Jersey 07103

**Keywords:** Alzheimer's disease, amyloid precursor protein (APP), β-amyloid (Aβ), presenilin, animal model, rat

## Abstract

Familial forms of Alzheimer's disease (FAD) are caused by mutations in the gene encoding amyloid precursor protein, whose processing can result in formation of β-amyloid (Aβ). FAD can also result from mutations in the *presenilin 1/2* (*PSEN1/2*) genes, whose protein products partially compose the γ-secretase complex that cleaves Aβ from amyloid precursor protein fragments. *Psen1* KO mice and knock-in (KI) mice with homozygous FAD-associated L435F mutations (*Psen1^LF/LF^*) are embryonic and perinatally lethal, precluding a more rigorous examination of the effect of Alzheimer's disease–causing *Psen1* mutations on neurodegeneration. Given that the rat is a more suitable model organism with regard to surgical interventions and behavioral testing, we generated a rat KI model of the *Psen1^LF^* mutation. In this study, we focused on young *Psen1^LF^* rats to determine potential early pathogenic changes caused by this mutation. We found that, unlike *Psen1^LF/LF^* mice, *Psen1^LF/LF^* rats survive into adulthood despite loss of γ-secretase activity. Consistent with loss of γ-secretase function, *Psen1^LF/LF^* rats exhibited low levels of Aβ38, Aβ40, and Aβ42 peptides. In contrast, levels of Aβ43, a longer and potentially more amyloidogenic Aβ form, were significantly increased in *Psen1^LF/LF^* and *Psen1^LF/w^* rats. The longer survival of these KI rats affords the opportunity to examine the effect of homozygous *Psen1* Alzheimer's disease–associated mutations on neurodegeneration in older animals.

## Introduction

Familial Alzheimer's disease (FAD)^2^ is caused by mutations in *PSEN1* and *PSEN2*, with the majority occurring in *PSEN1* (1). These genes encode Presenilin 1 (PS1) and Presenilin 2 (PS2), members of the γ-secretase complex (2, 3). FAD-causing mutations occur at hundreds of different loci over the span of *PSEN1* (RRID: SCR_006416), and the biochemical effect of *PSEN1* mutations is complex. In general, *PSEN1* mutations result in a decrease in endopeptidase activity and altered γ-processivity, resulting in reduced amounts of γ-secretase products and a relative increase in the longer forms of γ-secretase products. With regard to amyloid precursor protein (APP) processing, *PSEN1* mutations show reduced levels of β-amyloid (Aβ) (4), and some but not all mutations show a relative increase in longer forms of Aβ (5), whose accumulation is seen in FAD. In addition to the diverse effects on metabolite levels of a single substrate, γ-secretase has multiple substrates (6) whose function may impact neurodegenerative and neurodevelopmental processes in manner unrelated to the neurodegeneration caused by Aβ. Knock-in mouse (7) and *in vitro* models (8) of the FAD-causing *PSEN1 L435F* mutation show near-complete abrogation of γ-secretase activity and a reduction in total amyloid production. There are reports of a relative increase in Aβ43, a longer and potentially more amyloidogenic form of Aβ, in *PSEN1 L435F* FAD brains (9) and cell lines (9, 10) expressing PS1-L435F, but the absolute amount of Aβ43 produced is low and, in the case of KI mouse models (7), undetectable. The *PSEN1 L435F* mutation has not been studied in homozygosis, as *Psen1 L435F* homozygote mice are perinatally lethal (7) in a manner that resembles the early embryonic lethality of *Psen1* KO mice (11), likely the result of PS1 L435F-mediated disruption of Notch signaling. Given this lethality, the *Psen1 L435F* mutation was characterized in heterozygosis on the *Psen2* KO background to eliminate compensation from PS2 (7). Analysis of heterozygote *Psen1 L435F, Psen2-KO* mice showed marked synaptic memory deficits and an age-dependent neurodegenerative phenotype (7). Here we create a rat knock-in model of the *Psen1 L435F* mutation in a rat that expresses APP in which the Aβ region has been humanized (*Psen1^LF^* rats). A CRISPR/Cas9-mediated knock-in system was chosen to avoid the artifacts induced by the transgenic approach (*i.e.* nonphysiological overexpression, use of nonendogenous and/or non-cell-type-specific regulatory elements, and disruption of endogenous genes at integration sites). The rats were placed on a humanized APP background (12) to accommodate the possibility of differences in pathogenicity of rodent and human Aβ. Consistent with the mouse KI model, we found loss of γ-secretase function in *Psen1^LF/LF^* rats, which show minimal levels of Aβ38, Aβ40, and Aβ42 peptides; in contrast, concentrations of Aβ43 were significantly increased in *Psen1^LF/LF^* and *Psen1^LF/w^* rats. Unexpectedly, we also found that homozygote *Psen1^LF^* rats are born at Mendelian ratios, survive into adulthood, and have preserved neurodevelopment and Notch signaling despite altered APP metabolism. *Psen1^LF^* rats may therefore be a useful model for examination of neurodegenerative changes caused by *PSEN1 L435F* mutation.

## Results

### Generation of Psen1^LF^ rats carrying humanized App alleles (App^h/h^)

F0-*Psen1^LF^* rats were crossed to Long-Evans rats to generate F1-*Psen1^LF/w^* rats. These crossings were repeated four more times to obtain F5-*Psen1^LF/w^* rats. The probability that F5 rats carry unidentified off-target mutations (except those, if present, on chromosome 6) is ∼1.5625%. To generate *Psen1^LF^* rats on a background where rat App has a humanized Aβ region, F5-*Psen1^LF/w^* and *App^h/h^* rats were crossed to generate F1-*Psen1^LF/w^*; *App^h/w^* rats. The *App^w^* allele was removed in subsequent crosses. For all data generated in this study, all rats were on the *App^h/h^* background, which produces human and not rodent Aβ species.

**Figure 1.**
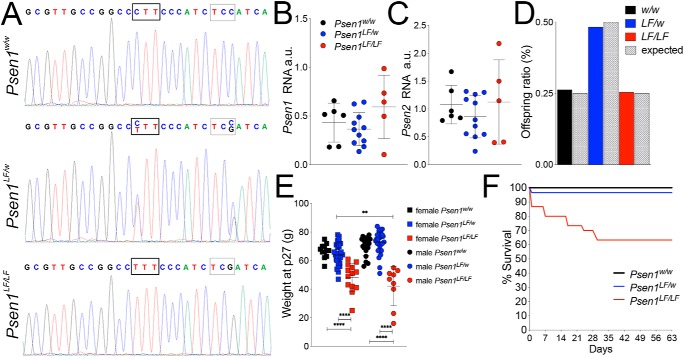
**Generation and survival of *Psen1^LF^* rats.** *A*, Sanger sequencing of PCR products from genomic DNA of *Psen1^w/w^*, *Psen1^LF/w^*, and *Psen1^LF/LF^* rats. *Thick-bordered boxes* denote the CTT→TTT codon change that defines the L435F mutation. *Thin-bordered boxes* denote a TCC→TCG silent mutation present in *Psen1^LF^* rats. *B*, levels of *Psen1* RNA, expressed as arbitrary units *a.u.*, from brain lysate were measured in *Psen1^w/w^*, *Psen1^LF/w^*, and *Psen1^LF/LF^* P0 rats by quantitative RT-PCR and normalized to *Gapdh* levels. No significant differences between *Psen1^w/w^*, *Psen1^LF/w^*, and *Psen1^LF/LF^* rats were evident. Data are represented as mean ± S.D. Data were analyzed by ordinary one-way ANOVA. *n* ≥ 5 rats/genotype. *C*, levels of *Psen2* RNA from brain lysate were measured in *Psen1^w/w^*, *Psen1^LF/w^*, and *Psen1^LF/LF^* P0 rats by quantitative RT-PCR and normalized to *Gapdh* levels. No significant differences between *Psen1^w/w^*, *Psen1^LF/w^*, and *Psen1^LF/LF^* rats were evident. Data are represented as mean ± S.D. Data were analyzed by ordinary one-way ANOVA. *n* ≥ 5 rats/genotype. *D*, genotype ratios at live birth of *Psen1^LF^* rats; *n* = 110. *E*, weight of rats at day 27. Data are represented as mean ± S.D. Data were analyzed by ordinary one-way ANOVA followed by post hoc Tukey's multiple comparisons test when ANOVA showed statistically significant differences. **, *p* < 0.01; ****, *p* < 0.0001. Numbers of each genotype: 12 female *Psen1^w/w^*, 29 female *Psen1^LF/w^*, 13 female *Psen1^LF/LF^*, 20 male *Psen1^w/w^*, 27 male *Psen1^LF/w^*, and 9 male *Psen1^LF/LF^. F*, survival curve of *Psen1^w/w^*, *Psen1^LF/w^*, and *Psen1^LF/LF^* rats from birth to day 28; *n* = 110 total.

To verify that the *Psen1^LF^* mutations were correctly inserted into *Psen1* exon 12, we amplified, by PCR, *Psen1* gene exon 12 from *Psen1^w/w^*, *Psen1^LF/w^*, and *Psen1^LF/LF^* rats. Sequencing of the PCR products showed that the mutations were correctly inserted in the *Psen1^LF/w^* and *Psen1^LF/LF^* genomes (Fig. 1*A*). RT-PCR analysis performed on RNA from P0 rat brain lysate from *Psen1^w/w^*, *Psen1^LF/w^*, and *Psen1^LF/LF^* rats confirmed that no alterations in *Psen1* or *Psen2* expression were caused by the *L435F* mutation (Fig. 1, *B* and *C*) (*Psen1*: F(2,19) = 1.864, *p* = 0.1824; *Psen2*: F(2,19) = 0.6833, *p* = 0.5169). KI *Psen1^LF/LF^* mice display perinatal lethality (7) and developmental abnormalities consistent with *Psen1* KO mice (11). To determine the lethality of the *L435F* mutation in KI rats, *Psen1^w/w^*, *Psen1^LF/w^*, and *Psen1^LF/LF^* rats were genotyped at birth, weighed at weaning, and followed for several weeks. *Psen1^w/w^*, *Psen1^LF/w^*, and *Psen1^LF/LF^* rats were born at Mendelian ratios (Fig. 1*D*). At the time of weaning (P27), male and female *Psen1^LF/LF^* rats weighed significantly less than *Psen1^w/w^* and *Psen1^LF/w^* littermates (Fig. 1*E*) (F(5,104) = 30.53, *p* < 0.0001; post hoc Tukey's multiple comparisons test results are reported in the figures). *Psen1^LF/LF^* rat survival declined to 65% by day 28 and then stabilized into adulthood, whereas *Psen1^w/w^* and *Psen1^LF/w^* littermates showed no significant postnatal lethality in the same period (Fig. 1*F*).

### Presenilinase activity, a prerequisite for γ-secretase function, is reduced in Psen1^LF^ rat brains

**Figure 2.**
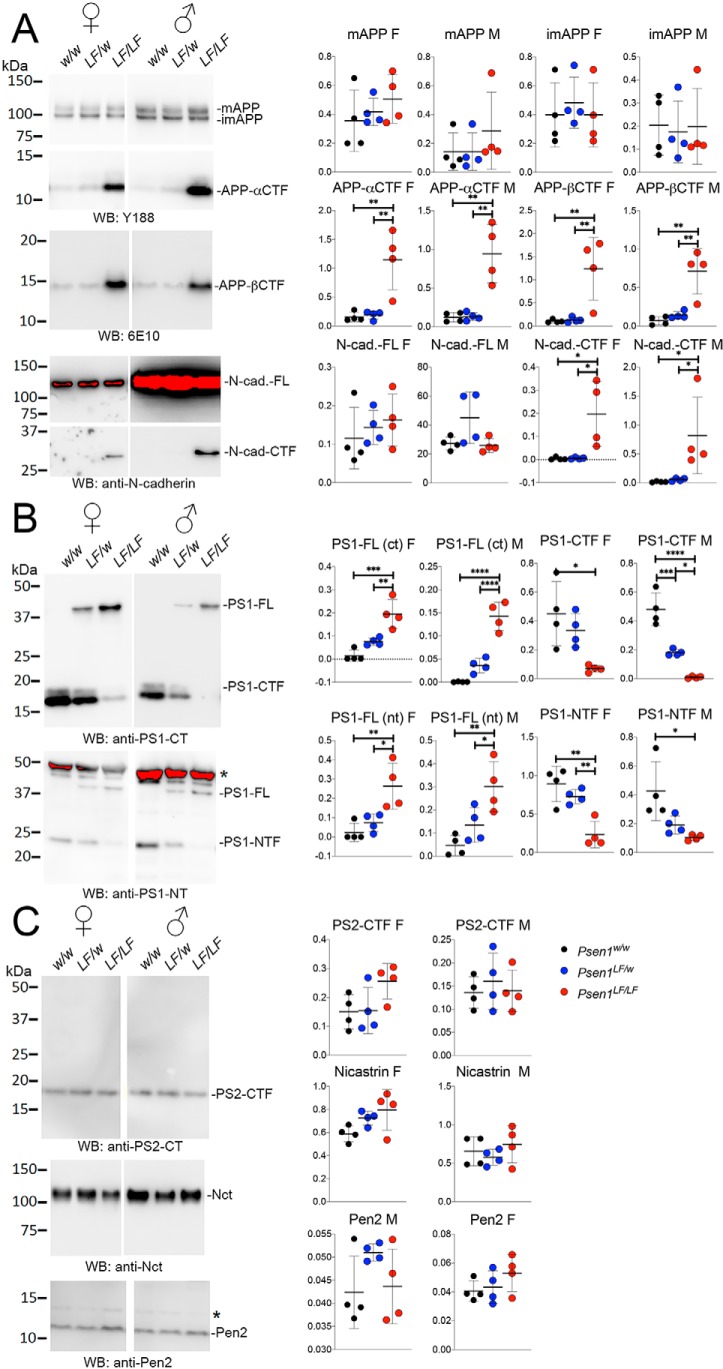
**Western blot analysis of γ-secretase substrates and components in *Psen1^LF^* rats.** Brain lysate of rat pups from male and female in *Psen1^w/w^*, *Psen1^LF/w^*, and *Psen1^LF/LF^* p4 rats were subjected to Western blot analysis with the following antibodies. *A*, Y188 APP C terminus (mature and immature full-length APP and APP-CTFs, predominantly APP αCTF, are indicated), 6E10 APP Aβ3–8 epitope (mature and immature full-length are oversaturated in this exposure and not quantified; APP βCTF is indicated), and N-cadherin C terminus (full-length N-cadherin and N-cadherin CTFs are indicated; the full-length N-cadherin signal is saturated). A lower, nonsaturated exposure was used for quantitation and is provided in Fig. S1. In Fig. S1, we show all the Western blot (*WB*) images used for quantitative analysis. *B*, Presenilin 1 C terminus and N terminus; full-length PS1 (detected by either an antiPS1-CT or and antiPS1-NT antibody), PS1-CTFs, and PS1-amino-terminal fragments are indicated. *C*, analysis of the γ-secretase components Presenilin 2 (using an antibody specific for the CT of PS2), Nicastrin, and Pen2; as for PS2, only the PS2-CTFs are indicated, as no holoenzyme is detectable. *Asterisks* at the right of each blot indicate nonspecific bands. Quantifications are presented for each set of Western blots. Data are represented as mean ± S.D. Data were analyzed by ordinary one-way ANOVA followed by post hoc Tukey's multiple comparisons test when ANOVA showed statistically significant differences. *n* = 4 rats/sex per genotype. *, *p* < .05; **, *p* < .01; ***, *p* < 0.001; ****, *p* < 0.0001.

As noted above, γ-secretase has several substrates, including APP and N-cadherin (13). Typically, γ-secretase mediates intramembranous cleavage of C-terminal fragments derived by prior processing by α- or β-secretase. As for APP, α- and β-secretase generate two γ-secretase substrates, APP-αCTF and APP-βCTF, respectively. α-Secretase cleavage of N-cadherin yields the γ-secretase substrate N-cad–CTF. Thus, to assess γ-secretase function in *Psen1^LF^* rats, solubilized brain lysate from P4 *Psen1^w/w^*, *Psen1^LF/w^*, and *Psen1^LF/LF^* pups was analyzed by Western blotting for steady-state levels of these γ-secretase substrates. *Psen1^LF/LF^* rat brains showed a sex-independent increase in APP-αCTF, APP-βCTF, and N-cad–CTF, whereas *Psen1^LF/w^* rat brains were indistinguishable from WT controls (Fig. 2*A*) (APP-αCTF F: F(2,9) = 13.43, *p* = 0.0020; APP-αCTF M: F(2,9) = 18.03, *p* = 0.0007; APP-βCTF F: F(2,9) = 10.78, *p* = 0.0041; APP-βCTF M: F(2,9) = 16.17, *p* = 0.0010; N-cad–CTF F: F(2,9) = 7.415, *p* = 0.0125; N-cad–CTF M: F(2,9) = 5.597, *p* = 0.0263; post hoc Tukey's multiple comparisons test results are reported in the figures). Full-length APP and N-cadherin were not significantly changed (Fig. 2*A*) (mature APP F: F(2,9) = 0.8284, *p* = 0.4675; mature APP M: F(2,9) = 0.7954, *p* = 0.4807; immature APP F: F(2,9) = 0.2181, *p* = 0.8082; immature APP M: F(2,9) = 0.0478, *p* = 0.9534; N-cad–FL F: F(2,9) = 0.5231, *p* = 0.6096; N-cad–FL M: F(2,9) = 3.778, *p* = 0.0644). Analysis of PS1 showed a sex-independent and *Psen1^LF^* allele dose-dependent decrease in autocatalysis of PS1 (*i.e.* Presenilinase activity), a prerequisite for γ-secretase function (14), as shown by the increase in full-length PS1 and decrease in PS1-CTFs and PS1-amino-terminal fragments in *Psen1^LF/w^* and *Psen1^LF/LF^* rat brains (Fig. 2*B*) (PS1-FL (COOH-terminal antibody) F: F(2,9) = 21.48, *p* = 0.0004; PS1-FL (COOH-terminal antibody) M: F(2,9) = 55.19, *p* < 0.0001; PS1-CTF F: F(2,9) = 7.236, *p* = 0.0134; PS1-CTF M: F(2,9) = 50.85, *p* < 0.0001; PS1-FL (NH3-terminal antibody) F: F(2,9) = 10.27, *p* = 0.0048; PS1-FL (NH3-terminal antibody) M: F(2,9) = 10.36, *p* = 0.0046; post hoc Tukey's multiple comparisons test results are reported in the figures). PS2 was not affected in a similar manner, nor were there any alterations in the levels of other members of the γ-secretase complex, Nicastrin and Pen2 (Fig. 2*C*) (PS2-CTF F: F(2,9) = 3.135, *p* = 0.0926; PS2-CTF M: F(2,9) = 0.2981, *p* = 0.7493; Nicastrin F: F(2,9) = 3.360, *p* = 0.0813; Nicastrin M: F(2,9) = 0.8258, *p* = 0.4685; Pen2 F: F(2,9) = 1.497, *p* = 0.2746; Pen2 M: F(2,9) = 1.193, *p* = 0.71920).

### γ-Secretase activity and processivity is reduced in Psen1^LF^ rat brains, increasing the ratio of long Aβ peptides/short Aβ peptides

**Figure 3.**
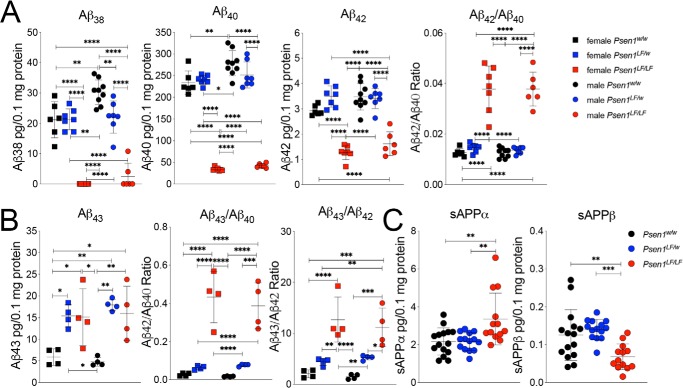
**ELISA measurements of amyloid species and soluble APP species in *Psen1^LF^* rats.** *A*, ELISA levels of Aβ38, Aβ40, and Aβ42 in male and female *Psen1^w/w^*, *Psen1^LF/w^*, and *Psen1^LF/LF^* P4 rat brain lysate. The ratio of Aβ42/Aβ40 is also presented. We used the following numbers of samples: *Psen1^w/w^* females *n* = 6, *Psen1^LF/w^* females *n* = 7, *Psen1^LF/LF^* females *n* = 7, *Psen1^w/w^* males *n* = 6, *Psen1^LF/w^* males *n* = 7, and *Psen1^LF/LF^* males *n* = 6. *B*, ELISA levels of Aβ43 in *Psen1^w/w^*, *Psen1^LF/w^*, and *Psen1^LF/LF^* P4 rat brain lysate. The ratios of Aβ43/Aβ40 and Aβ43/Aβ42 are also presented. Samples used: *n* = 4/sex/genotype. Aβ43 was quantified with the IBL Human Amyloidβ (1–43) (FL) Assay Kit (27710), validated by us using a rat *App* hypomorph control as shown in Fig. S2. *C*, ELISA levels of sAPPα and sAPPβ in *Psen1^w/w^*, *Psen1^LF/w^*, and *Psen1^LF/LF^* P4 rat brain lysate. Samples used: *Psen1^w/w^* females *n* = 6, *Psen1^LF/w^* females *n* = 7, *Psen1^LF/LF^* females *n* = 7, *Psen1^w/w^* males *n* = 6, *Psen1^LF/w^* males *n* = 7, and *Psen1^LF/LF^* males *n* = 6. Data are represented as mean ± S.D. Data were analyzed by ordinary one-way ANOVA followed by post hoc Tukey's multiple comparisons test when ANOVA showed statistically significant differences. *, *p* < 0.05; **, *p* < 0.01; ***, *p* < 0.001; ****, *p* < 0.0001.

Cleavage of APP-βCTFs by γ-secretase generates Aβ peptides, which vary in length depending on the processivity of γ-secretase; reduced processivity increases the relative amounts of longer Aβ peptides compared with shorter Aβ peptides. To complete the assessment of γ-secretase function in *Psen1^LF^* rats, solubilized brain lysates from *Psen1^w/w^*, *Psen1^LF/w^*, and *Psen1^LF/LF^* pups were analyzed by ELISA. Solubilized lysate was chosen for analysis, as the rats showed no insoluble Aβ plaques by immunohistochemistry (Fig. 5*E*). *Psen1^LF/LF^* rat brains had lower levels of Aβ38, Aβ40, and Aβ42 in a sex-independent manner (Fig. 3*A*) (Aβ38: F(5,36) = 59.20, *p* < 0.0001; Aβ40: F(5,36) = 183.8, *p* < 0.0001; Aβ42: F(5,36) = 41.91; post hoc Tukey's multiple comparisons test results are reported in the figures). *Psen1^LF/w^* animals in general had similar amyloid levels compared with WT rats, with the exception of lower levels of Aβ38 in female *Psen1^LF/w^* rats (Fig. 3*A*). Notably, the Aβ42/Aβ40 ratio was also increased in *Psen1^LF/LF^* rat brain lysates in a sex-independent manner (Fig. 3*A*) (F(5,36) = 49.06, *p* < 0.0001). Overall, the decrease in γ-secretase products (Aβ peptides), increase in γ-secretase substrates (APP-βCTF, APP-αCTF, and N-cad–CTF), and decrease in the autocatalysis of PS1 in *Psen1^LF^* rats is indicative of loss of γ-secretase function.

Although Aβ40 and Aβ42 levels were decreased in *Psen1^LF/LF^* rat brains, an absolute increase in Aβ43 was evident in soluble brain lysate from *Psen1^LF/w^* and *Psen1^LF/LF^* animals, male and female (Fig. 3*B*) (F(5,18) = 8.119, *p* = 0.00004). We also detected an increase in relative levels of Aβ43/Aβ40 and Aβ43/Aβ42 ratios (Fig. 3*B*) Interestingly, only one allele with the *Psen1^LF^* mutation was sufficient to drive the increase in Aβ43 levels, whereas the Aβ43/Aβ40 and Aβ43/Aβ42 ratios showed a trend of gene dose dependence and significant gene dose dependence, respectively (Aβ43/Aβ40: F(5,18) = 26.70, *p* < 0.0001; Aβ43/Aβ42: F(5,18) = 15.15, *p* < 0.0001). Thus, the L435F mutation also reduces γ-secretase processivity *in vivo*, as indicated by the absolute (Aβ43) and relative (Aβ42) increases in longer Aβ peptides.

To assess α- and β-secretase cleavage of APP, solubilized brain lysates were analyzed by ELISA for soluble APP ectodomain levels, sAPPα and sAPPβ, the other products of α- and β-cleavage of APP, respectively. No differences were seen in *Psen1^LF/w^* rat brains compared with WT rats (Fig. 3*B*). Surprisingly, *Psen1^LF/LF^* rat brain lysates show an increase in sAPPα levels and a decrease in sAPPβ levels (Fig. 3*B*) (sAPPα: F (2. 39) = 7.513, *p* = 0.0017; sAPPβ: F (2. 39) = 8.909, *p* = 0.0007), indicative of a secondary increase in α-secretase and decrease in β-secretase activity caused by the pathogenic *Psen1* mutation.

### PS1-L435F forms a γ-secretase complex

**Figure 4.**
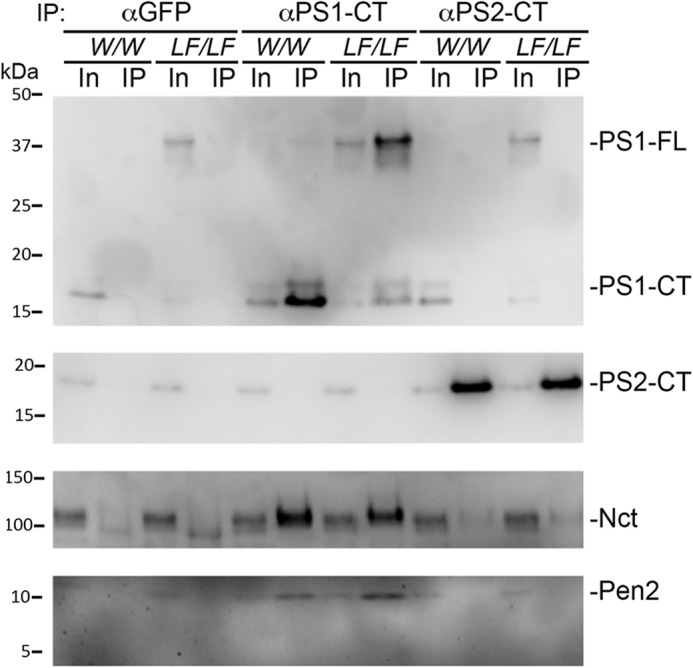
**Immunoprecipitation of γ-secretase complexes from *Psen1^w/w^* and *Psen1^LF/LF^* rats.** 1% CHAPSO–solubilized membrane from *Psen1^w/w^* and *Psen1^LF/LF^* rat brains was immunoprecipitated and analyzed by Western blotting. γ-Secretase complexes were immunoprecipitated with anti-PS1 or anti-PS2, and anti-GFP was used as a control. Input (*In*) and eluate (*IP*) are shown. PS1-WT and PS1-L435F specifically bind Nicastrin (*Nct*) and Pen2.

Given the overall trend toward *Psen1^LF^* conferring a loss-of-function phenotype, Aβ43 levels notwithstanding, we wished to determine whether loss of function was the result of the inability of PS1-L435F to form an active γ-secretase complex. Anti-PS1 and anti-PS2 antibodies were used to immunoprecipitate γ-secretase complexes from 1% CHAPSO–solubilized brain lysate from *Psen1^w/w^* and *Psen1^LF/LF^* rats (Fig. 4). *Psen1^w/w^* and *Psen1^LF/LF^* samples coimmunoprecipitated Nicastrin and Pen2 when immunoprecipitated with anti-PS1, indicating that PS1-L435F forms a γ-secretase complex. Anti-PS2 immunoprecipitated PS2-containing complexes, as seen by the presence of Nicastrin in the eluate, but Pen2 levels were below the level of detection.

### Notch signaling is not significantly impaired in Psen1^LF/LF^ rats

**Figure 5.**
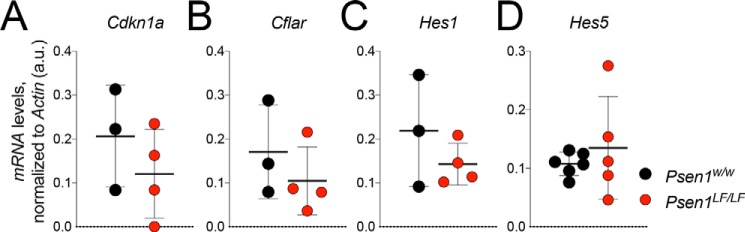
**RT-PCR analysis of Notch intracellular domain target genes in *Psen1^LF^* rats.** *A–D*, expression levels of several Notch intracellular domain target genes were evaluated in RNA derived from P0 rat brain lysate from *Psen1^w/w^* and *Psen1^LF/LF^* pups. Expression of *Cdkn1a* (*A*), *Cflar* (*B*), and *Hes1* and *Hes5* (*C* and *D*) showed no significant differences between *Psen1^w/w^* and *Psen1^LF/LF^* rats. Data are represented as mean ± S.D. Data were analyzed by ordinary one-way ANOVA. Samples used: for *Cdkn1a*, *CASP8*, *Cfla*, and *Hes1*, *Psen1^w/w^ n* = 3 and *Psen1^LF/LF^ n* = 4; for *Hes5*, *Psen1^w/w^ n* = 6 and *Psen1^LF/LF^ n* = 5.

Impaired neurogenesis in *Psen1^LF/LF^* mice is coincident with disrupted Notch signaling. To determine the status of Notch signaling in *Psen1^LF/LF^* rats, expression levels of several Notch intracellular domain target genes were analyzed by RT-PCR. Analysis of cyclin-dependent kinase inhibitor 1A (*Cdkn1a*), CASP8 and FADD-like apoptosis regulator (*Cflar*), and hairy and enhancer of split 1 and 5 (*Hes1* and *Hes5*, respectively) levels in RNA derived from P0 *Psen1^w/w^* and *Psen1^LF/LF^* rat brain lysates showed no significant differences (Fig. 5, *A–D*) (two-tailed unpaired *t* test; *Cdkn1a p* = 0.3417, *Cflar p* = 0.3838, *Hes1 p* = 0.3124, *Hes5 p* = 0.4719). This result suggests that Notch signaling is not impaired in *Psen1^LF/LF^* rats.

### Psen1^LF^ rat brains show no neurodevelopmental or histopathological changes at day 15

**Figure 6.**
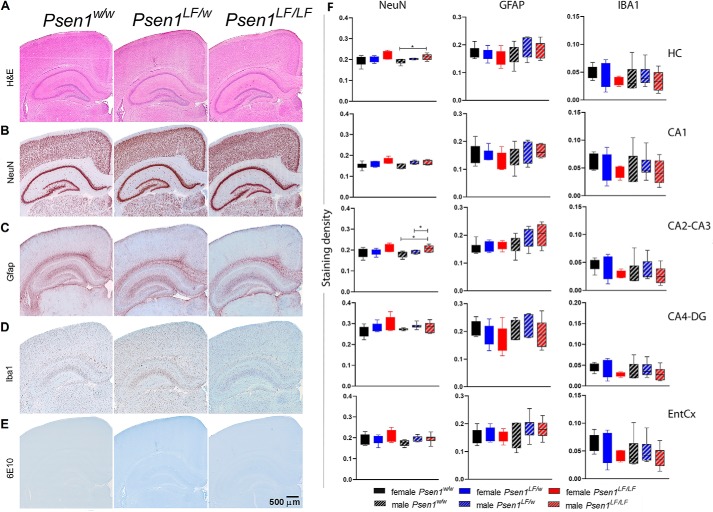
**Histopathological analysis of *Psen1^LF^* rats.** IHC analysis of P15 rat whole hippocampus (*HC*), hippocampal subregions (*CA1*, *CA2–CA3*, and *CA4-DG*), or entorhinal cortex (*EntCx*). *A*, H&E-stained sections from *Psen1^w/w^*, *Psen1^LF/w^*, and *Psen1^LF/LF^* rats. *B*, evaluation of neuronal number by NeuN staining, with quantitation of total NeuN intensity on the *right. n* ≥ 5 rats/sex/genotype. Data were analyzed by one-way ANOVA with Kruskal-Wallis post-test; *, *p* < 0.05. Data are represented as mean ± S.D. *C*, evaluation of astrogliosis by Gfap staining, with quantitation of total Gfap intensity on the *right*. Data were analyzed by one-way ANOVA. Data are represented as mean ± S.D. *D*, evaluation of microgliosis by Iba1 staining, with quantitation of total Iba1 intensity on the *right. n* ≥ 5 rats/sex/genotype. Data were analyzed by one-way ANOVA. Data are represented as mean ± S.D. *E*, evaluation of amyloid plaques by 6E10 staining.

To determine whether the biochemical changes caused by the *Psen1 L435F* mutation impact neurodevelopment or cause neuropathology, we used histology and immunohistochemistry (IHC) analysis to characterize brains from p15 *Psen1^w/w^*, *Psen1^LF/w^*, and *Psen1^LF/LF^* rats. Regions of analysis included the frontal cortex, cingulate cortex, whole hippocampus, and entorhinal cortex. No gross morphological changes were evident by H&E staining in any of the rats analyzed (Fig. 6*A*). Qualitative inspection of NeuN staining showed no appreciable changes in neuronal density in any of the regions analyzed in *Psen1^LF^* rats, but a quantitative analysis of total NeuN signal found a small but statistically significant increase in male *Psen1^LF/LF^* rat whole hippocampus and the CA2–CA3 region (Fig. 6*B*). No evidence of astrocytosis or microgliosis was seen by staining with Glial fibrillary acidic protein and IBA1, respectively, in any of that rats tested (Fig. 6, *C* and *D*). Amyloid plaques, as measured by staining the anti-Aβ antibody 6E10, were absent in all rats tested (Fig. 6, *E* and *F*). Overall, histological analysis of these rats shows no evidence of neurodevelopmental impairments or FAD-like pathology at 15 days.

## Discussion

The choice of animal model and genetic approach have profound implications on the phenotypic expression of disease-associated mutations. Given the better suitability of rats for behavioral tests, surgical procedures, and the expression of tau isoforms that more closely reflects human tau splicing, we chose to model FAD-related mutations using a Long-Evans rat KI model (12). The *Psen1 L435F* mutation was selected, given its profound alteration of APP metabolism (8) and age-dependent neurodegenerative changes seen in KI Psen1*^LF^* mice (7). Here we studied young *Psen1^LF^* rats to determine potential early pathogenic mechanisms caused by this pathogenic mutation. Unexpectedly, we found that, in contrast to *Psen1^LF/LF^* mice, *Psen1^LF/LF^* rats survive into adulthood. This survival is likely the result of the Notch-sparing phenotype seen in *Psen1^LF/LF^* rats that is absent in *Psen1^LF/LF^* KI mice. Three nonmutually exclusive possibilities may underlie this Notch-sparing effect. 1) PS1-L435F can assemble in a γ-secretase complex (Fig. 4). Given the profound decrease in processing of APP and N-cadherin in *Psen1^LF^* rats, it is unlikely, but still formally possible, that the mutant PS1 is catalytically active in rats in a substrate-specific manner. This possibility would be in line with previous data showing that FAD mutant PS1 is able to rescue the Notch phenotype independent of the APP pathway (16). 2) It is also possible that, given the reduction in autocatalysis, PS1-L435F changes localization and is sequestered in the cell so that is exposed to different substrates than PS1 WT. 3) There may be a partial compensation of catalytically inactive PS1-L435F by PS2 (17). Rat *Psen2* may be expressed earlier compared with mouse *Psen2* during embryonic development. At P0, this compensation would necessarily be a qualitative change in localization/activity and not a difference in quantity, as *Psen2* expression and PS2 levels are unchanged in *Psen1^LF/LF^* rats.

Amyloid levels vary considerably between models. Although Aβ40 and Aβ42 levels were undetectable in *Psen1^LF/LF^* mice, both species were detected in this study at about 9% and 40% of WT controls for Aβ40 and Aβ42, respectively. In general, cell culture and *in vitro* models show that PS1 L435F mediates loss of Aβ40 and Aβ42 production. PS1 L435F–reconstituted PS1/2 KO mouse embryonic fibroblasts (10) demonstrate undetectable Aβ40 levels and a more than 90% reduction in Aβ42, whereas stably transfected PS1-L435F HEK cells (9) show a more than 90% reduction in both species, but it must be considered that these cell lines overexpress APP and PS1, and therefore no inference can be made regarding the absolute levels of Aβ production. In liposome-based *in vitro* assays of recombinant PS1 L435F activity (4), PS1 L435F γ-secretase activity, as measured by Aβ40 and Aβ42, was found to be nearly undetectable, at 0.007 times the activity of WT PS1. Measurement of Aβ43 has similarly varied across models and groups, with *in vitro* overexpression models of PS1-L435F activity demonstrating an increase in the relative amounts of Aβ43 (9, 10), a finding not recapitulated in *Psen1^LF^* KI mice (7). Our models revealed an absolute increase in Aβ43 and Aβ42/40 and Aβ43/40 ratios in KI rats, but these observations occurred in the setting of decreased total Aβ and no apparent Aβ aggregation. How well these models relate to amyloid metabolism of *PSEN1 L435F* FAD patients is unclear, as total amyloid levels have not been determined in autoptic brain tissue, but aggregated forms of Aβ42 and Aβ43 are present in histopathological analysis (9).

Use of the KI system, in which endogenous APP is expressed, allows more complete analysis of APP metabolism beyond Aβ. APP-CTFs are the direct substrate of PS1/2, and βCTFs are expectedly increased in *Psen1^LF/LF^* rats concurrent with Aβ reduction. This increase in APP-CTFs may have a pathogenic effect *per se* (18–21). In addition, *Psen1^LF/LF^* rats show an effect on the metabolism of full-length APP as well. Specifically, there is a significant increase in sAPPα and a significant decrease in sAPPβ, indicative of a shift toward α processing of APP. Coordination between γ and α/β processing is possible, given recent evidence that a fraction of γ-secretase exists in a tripartite macromolecular complex with APP and ADAM10 (22) or BACE1 (23). A stalled or otherwise inactivated PS1-L435F–containing γ complex may differentially affect the complex's ability to bind ADAM10 or BACE1. Apart from the potential general impact PS1-L435F may have on α/β -secretase, the finding of increased sAPPα and decreased sAPPβ is significant by itself, as these and other non-Aβ metabolites of APP have been implicated as modulators of synaptic activity (12, 24–26) and neuronal survival (27).

Although the IHC analysis of day 15 *Psen1^LF^* rats is consistent with normal neurodevelopment, there is also no indication of Aβ plaques, astrogliosis, or microgliosis that occur in FAD. The lack of amyloid pathology at day 15 is unsurprising, considering that even in animal models in which APP with FAD-related mutations is overexpressed, plaques take at least 6 weeks to develop (28). *Psen1^LF^* rats may require extensive aging or additional mutations to develop FAD-related histopathological changes; however, given the survival of *Psen1^LF/LF^* rats and avoidance of a Notch-related phenotype, the *Psen1^LF^* rat KI model is a useful, physiologically appropriate model with which to study age-related neurodegeneration in FAD.

## Experimental procedures

### Rats and ethics statement

Rats were handled according to the Ethical Guidelines for Treatment of Laboratory Animals of the National Institutes of Health. The procedures were approved by the Institutional Animal Care and Use Committee at Rutgers.

### Generation of rats expressing the FAD Psen1 L435F mutation (Psen1^LF^ rats)

The rat *Psen1* gene (GenBank: NM_019163; Ensembl: ENSRNOG00000009110) is located on rat chromosome 6. We created a Long-Evans rat model with point mutation CTT→TTT at the rat *Psen1* locus by CRISPR/Cas-mediated genome engineering. This mutation creates a rat that carries a *Psen1* gene coding for PS1 with the FAD L435F mutation. The detailed procedures are reported in the Supporting Experimental Procedures.

### Rat brain preparation

Rats were anesthetized with isoflurane and perfused via intracardiac catheterization with ice-cold PBS. Brains were extracted and homogenized using a glass–Teflon homogenizer (100 mg tissue/1 ml buffer (w/v)) in 250 mm sucrose, 20 mm Tris-base (pH 7.4), 1 mm EDTA, and 1 mm EGTA plus protease and phosphatase inhibitors (Thermo Scientific), with all steps carried out on ice or at 4 °C. Total lysate was solubilized with 0.1% SDS and 1% NP-40 for 30 min while rotating. Solubilized lysate was spun at 20,000 × *g* for 10 min, the supernatant was collected and analyzed by ELISA and Western blotting.

### Western blot analysis

Biochemical analysis of rat brain samples was performed as described previously (29). Briefly, protein content was quantified by Bradford analysis prior to solubilization. 15 μg of protein was brought to 15 μl with PBS and LDS sample buffer (10% β-mercaptoethanol (Invitrogen, NP0007) and 4.5 m urea) to 1× and loaded on a 4%–12% BisTris polyacrylamide gel (Bio-Rad, 3450125). Proteins were transferred onto nitrocellulose at 25 V for 7 min using the Trans-blot Turbo system (Bio-Rad) and visualized by red Ponceau staining. Membranes were blocked for 30 min in 5% milk (Bio-Rad, 1706404) and washed extensively in PBS/Tween 20 (0.05%), and primary antibody was applied overnight at 4 °C at 1:1000 dilution in blocking solution (Thermo, 37573). The following antibodies were used: Tyr-188 (APP C terminus, Abcam, ab32136), 6E10 (APP Aβ3–8 epitope, Biolegend, 803001), Pen2 (Cell Signaling Technology, 8598), Presenilin 2 (Cell Signaling Technology, 2192), Nicastrin (Cell Signaling Technology, 5665), N-cadherin (Cell Signaling Technology, 14215), Presenilin 1 C terminus (Cell Signaling Technology, 5643), and Presenilin 1 N terminus (Biolegend, 811101). Anti-mouse (Southern Biotech, 1031-05) or a 1:1 mixture of anti-rabbit (Southern Biotech, OB405005) and anti-rabbit (Cell Signaling Technology, 7074), was diluted 1:1000 in 5% milk and used against mouse and rabbit primary antibodies for 30 min at room temperature with shaking. Blots were developed with West Dura ECL reagent (Thermo, PI34076) and visualized on a ChemiDoc MP Imaging System (Bio-Rad). Signal intensity was quantified with Image Lab software (Bio-Rad). Data were analyzed using Prism software and are represented as mean ± S.D.

### ELISA

For analysis of Aβ38, Aβ40, Aβ42, sAPPα, and sAPPβ, the following Meso Scale Discovery kits were used. Aβ38, Aβ40, and Aβ42 were measured with V-PLEX Plus Aβ Peptide Panel 1 6E10 (K15200G) and V-PLEX Plus Aβ Peptide Panel 1. sAPPα and sAPPβ were measured with sAPPα/sAPPβ (K15120E). Measurements were performed according to the manufacturer's recommendations. Plates were read on a Meso QuickPlex SQ 120. For analysis of Aβ43, the IBL Human Amyloidβ (1–43) (FL) Assay Kit (27710) was used according to the manufacturer's recommendations. The specificity of this kit was validated by us using a rat *App*^δ^*^7^*^/δ^*^7^* hypomorph control (12, 29) (validation data are presented in Fig. S2). Data were analyzed using Prism software and are represented as mean ± S.D.

### Immunoprecipitation

Total brain lysate was diluted in immunoprecipitation buffer (50 mm Tris, 150 mm NaCl, 1 mm EGTA, and 1 mm EDTA (pH 8.0)) with 1% CHAPSO, solubilized for 30 min at 4 °C while rotating, and spun at 20,000 × *g* for 10 min. Solubilized lysate was used as input for immunoprecipitation with anti-GFP (Cell Signaling Technology, 2555), anti-PS1-CT, or anti-PS2-CT antibodies and protein A/G beads (Thermo, 20421) overnight at 4 °C while rotating. After several wash steps, bound protein was eluted by 5-min incubation with 1× LDS sample buffer at 55 °C. Input and eluates were analyzed by Western blot analysis.

### RT-PCR

Total brain RNA was extracted with the RNeasy RNA Isolation kit (Qiagen) and used to generate cDNA with a High-Capacity cDNA Reverse Transcription Kit (Thermo). 50 ng of cDNA, TaqMan^TM^ Fast Advanced Master Mix (Thermo, 4444556), and the appropriate TaqMan (Thermo) probes were used in the real-time PCR. Samples were analyzed on a QuantStudio 6 Flex Real-Time PCR System (Thermo), and relative RNA amounts were quantified using LinRegPCR software. The probe Rn00570673_m1 (exon junctions 11–12, 12–13, and 13–14) was used to detect rat *Psen1*, and samples were normalized to *Gapdh* levels, as detected with Rn01775763_g1 (exon junctions 2–3 and 7–8). Levels of Notch target gene transcripts were determined using the RT^2^ Profiler^TM^ PCR Array Rat Notch Signaling Pathway plate (Qiagen, 330231 PARN-059Z) according to the manufacturer's recommendations. Student's *t* test was used for all analyses, with data presented as mean ± S.D.

### Immunohistochemistry (IHC)

#### 

##### Staining tissue preparation and sectioning

Rat brain tissue was prepared and stained as described previously. Briefly, intracardiac paraformaldehyde-perfused rat brains were extracted and stored in 70% ethanol prior to cerebral coronal sectioning. Sections were dehydrated and paraffin-embedded and then processed into 15 cross-sections targeting the frontal cortex at the level of the isthmus of the corpus callosum and anterior and posterior hippocampus. IHC staining was performed in accordance with Biospective Standard Operating Procedure BSP-L-06. Slides were manually deparaffinized and rehydrated prior to automated IHC. Slides initially underwent antigen retrieval, either heat-induced epitope retrieval or formic acid treatment. All IHC studies were performed at room temperature on a Lab Vision Autostainer using the REVEAL Polyvalent HRP-AEC Detection System (Spring Bioscience). Briefly, slides were incubated sequentially with hydrogen peroxide for 5 min to quench endogenous peroxidase, followed by 5 min in Protein Block, and then incubated with primary antibodies (Gfap, Thermo RB-087-A; NeuN, Millipore, A60; Aβ, 6E10 Biolegend; Iba1, Wako, 013-27691). Antibody binding was amplified using Complement reagent (20 min) followed by an HRP conjugate (20 min) and visualized using the AEC Chromogen (20 min). All IHC sections were counterstained with Acid Blue 129 and mounted with aqueous mounting medium (15).

##### Image analysis of IHC sections

The IHC and histology slides were digitized using an Axio Scan.Z1 digital whole-slide scanner (Carl Zeiss). The images underwent a quality control review, and final images were transferred to the Biospective server for qualitative image analysis. All qualitative assessments were performed blinded to the tissue genotype.

### Statistical analysis

Statistical significance was evaluated using ordinary one-way ANOVA followed by post hoc Tukey's multiple comparisons test when applicable (*i.e.* when the ordinary one-way ANOVA showed statistical significance). Statistical analysis was performed with GraphPad Prism v8 for Mac. Significant differences were accepted at *p* < 0.05.

### Data availability

All of the data are contained in the manuscript.

## Author contributions

M. D. T. and L. D. conceptualization; M. D. T. and L. D. data curation; M. D. T. and L. D. formal analysis; M. D. T. and L. D. validation; M. D. T. and L. D. investigation; M. D. T. and L. D. visualization; M. D. T. and L. D. methodology; M. D. T. and L. D. writing-original draft; M. D. T. and L. D. writing-review and editing; L. D. resources; L. D. supervision; L. D. funding acquisition.

## Supplementary Material

Supporting Information

## Footnotes

2 The abbreviations used are:

FAD: familial Alzheimer's disease
APP: amyloid precursor protein
Aβ: β-amyloid
KI: knock-in
P0: postnatal day 0
M: male
F: female
N-cad: N-cadherin
FL: full-length
CTF: carboxy-terminal fragment
CHAPSO: 3-[(3-cholamidopropyl)dimethylammonio]-2-hydroxy-1-propanesulfonic acid
IHC: immunohistochemistry
cDNA: complementary DNA
ANOVA: analysis of variance.
