# Supplementary material for: Knock-in rats with homozygous PSEN1L435F Alzheimer mutation are viable and show selective γ-secretase activity loss causing low Aβ40/42 and high Aβ43
Source: J Biol Chem. 2020 Apr 7;295(21):7442–51. doi: 10.1074/jbc.RA120.012542 (PMC7247318; doi:10.1074/jbc.RA120.012542)
Supplement: Supporting Information [file supp_295_21_7442__index.html]

Knock-in rats with homozygous PSEN1L435F Alzheimer mutation are viable and show selective γ-secretase activity loss causing low Aβ40/42 and high Aβ43 — Loss of γ-secretase activity in Presenilin-1 L435F KI rat — Supporting Information 

# Knock-in rats with homozygous *PSEN1L435F* Alzheimer mutation are viable and show selective γ-secretase activity loss causing low Aβ40/42 and high Aβ43

## Supporting Information

- Supporting Information (to be published online) - 1. Extended Experimental Procedures 2. Figure S1 &#x2013; Whole Western Blot images used in Figure 2 3. Figure S2 &#x2013; Validation of IBL Human Amyloid&#x03B2; (1-43) (FL) Assay Kit (27710) using a rat App hypomorph control
