# Supplementary material for: Knock-in rats with homozygous PSEN1L435F Alzheimer mutation are viable and show selective γ-secretase activity loss causing low Aβ40/42 and high Aβ43
Source: J Biol Chem. 2020 Apr 7;295(21):7442–51. doi: 10.1074/jbc.RA120.012542 (PMC7247318; doi:10.1074/jbc.RA120.012542)

Knock-in rats with homozygous *PSEN1*^{L435F} Alzheimer mutation are viable and show selective γ-secretase activity loss causing low Aβ40/42 but high Aβ43

Marc D. Tambini & Luciano D'Adamio

Supporting Information

1. Extended Experimental Procedures
2. Figure S1 – Whole Western Blot images used in Figure 2
3. Figure S2 – Validation of IBL Human Amyloidβ (1-43) (FL) Assay Kit (27710) using a rat *App* hypomorph control

1. Extended Experimental procedures

Generation of rats expressing the FAD *Psen1 L435F* mutation (*Psen1*^{LF} rats).

MTEIPAPLSYFQNAQMSEDSHSSSVRSQNDNQERQQHHDRQRLDNPESISNGRPQSNFTRQVIEQDEEEDEELTLKYGAKHVIMLF VPVTLCMVVVVATIKSVSFYTRKDGQLIYTPFTEDTETVGQRALHSILNAAIMISVIVVMTILLVVLYKYRCYKVIHAWLIVSSLLLLFFFSFIYLG EVFKTYNVAVDYITVALLIWNFGVVGMIAIHWKGPLRLQQAYLIMISALMALVFIKYLPEWTAWLILAVISVYDLVAVLCPKGPLRMLVETAQE RNETLFPALIYSSTMVWLVNMAEGDPEAQRRVPKNPKYSTQGTEREETQDTGTGSDDGGFSEEWEAQRDSHLGPHRSTPESRAAVQEL SGSILTSEDPEERGVKLGLGDFIFYSVLVGKASATASGDWNTTIACFVAILIGLCLTLLLLAIFKKALPAFPISITFGLIFYFATLAFHQFYI

Amino acid sequence of PS1-L435F. L→F mutation is in red.

The rat *Psen1* gene comprises 12 exons, with the ATG start codon in exon 3 and the TAG stop codon in exon 12. The CTT codon for L^{435} is located in exon 12 that was selected as target site. A silent mutation (TCC to TCG) will also be introduced to prevent the binding and re-cutting of the sequence by Cas9 after homology-directed repair. gRNA targeting vector and oligo donor (with targeting sequence, flanked by 120bp homologous sequences combined on both sides) were designed as follows.

Vector Type: Mammalian CRISPR Vector pRP[CRISPR]-hCas9-U6 (Single gRNA)
gRNA1: GTGATGGAGATGGGAAGGGCCGG
gRNA2: GAAAATGAGCCCGAAGGTGATGG

The links for a detailed description of the gRNA vectors can be found in the following sites:
gRNA1: http://www.vectorbuilder.com/design/report/336a28a7-0469-474a-baf0-f90e4952886c
gRNA2: http://www.vectorbuilder.com/design/report/897daed3-8c92-445b-9733-39456cbbcfa5
5'-CCACAGGGCCTGTGCCTTACGTTACTCCTGCTCGCCATTTTCAAGAAAGCGTTGCCGGCCCTTCCCATCTCCAT CACCTTCGGGCTCATTTTCTACTTTGCCACGGATTATCTCGTGCAGCCC-3'

3'-GGTGTCCCGGACACGGAATGCAATGAGGACGAGCGGTAAAAGTTCTTTCGCAACGGCCGGGAAGGGTAGAGGTA GTGGAAGCCCGAGTAAAAGATGAAACGGTGCCTAATAGAGCACGTCGGG-5'

Rat *Psen1* exon 12 targeted sequence. The gRNA1 is highlighted in yellow and gRNA2 is double underlined (the two sequences are partially overlapping). The nucleotides targeted for mutation are underlined.

5'-CCACAGGGCCTGTGCCTTACGTTACTCCTGCTCGCCATTTTCAAGAAAGCGTTGCCGGCCTTTCCCATCTCGATCA CCTTCGGGCTCATTTTCTACTTTGCCACGGATTATCTCGTGCAGCCC-3'

Oligo donor sequence, with the mutated nucleotide in red and yellow highlight and the silent TCC to TCG mutation in red.

Cas9 mRNA, gRNA generated by *in vitro* transcription and oligo donor were co-injected into zygotes for production of rats carrying these knock-in (KI) mutations by homology-directed repair. To verify CRISPR-induced mutation the pups were genotyped by PCR, followed by sequence analysis. The rat *Psen1* locus was amplified by PCR with the specific primers: F: TTGGTTGCGAGGCATCATGGTA and R: CCCAAGTCCAGTAGTGCAGGTGG. To identify founders carrying correctly targeted *Psen1* alleles, the 456 bp long PCR products were cloned into TA vectors and 10 plasmids containing *Psen1* inserts were sequenced using primer R: CCCAAGTCCAGTAGTGCAGGTGG. This analysis showed that founders Rat-ID#13 and Rat-ID#20 carried the mutant allele (WT= Wild-type):

Wildtype	5'-CGGCCCTTCCCATCTCCATCACCTTCGGGCTCATTTTCTACTTTG-3'
ID#13	5'-CGGCCTTTCCCATCTCGATCACCTTCGGGCTCATTTTCTACTTTG-3'
ID#20	5'-CGGCCTTTCCCATCTCGATCACCTTCGGGCTCATTTTCTACTTTG-3'

Sequence of the allele carrying the FAD mutation (boxed and in red) and the silent mutation (boxed and in blue) compared to the sequence of rat *Psen1*. The gRNA sequence is underlined

Thus, Rat-ID#13 and Rat-ID#20 were identified as a positive chimeric founder (F0-*Psen1*^{LF} rat). *Off-target analysis of targeting sequence* gRNA1**:** GAAAATGAGCCCGAAGGTGATGG**.** We identified five potential off-target sites for gRNA1. Based on this analysis, Rat-ID#13 and Rat-ID#20 have been analyzed for mutations in these most likely off-target mutation sites. Mismatched bases are in red. These sites have been amplified by PCR and sequenced.

Off-target site on chr 13: 98514253 AAAGATGAGCCCGAAGGTGATGG 985142

Targeting sequence: GAAAATGAGCCCGAAGGTGATGG

A 490bp products was generated by PCR with the following forward (F) and reverse (R) oligos:
F-CTCTGACGGGCAAGCCTCAG; R-GGCTGTTGCATATCTGTAAGAGACGA
The PCR product was sequenced using primer R. Sequencing results comparing WT rat and Rat-ID#13 and Rat-ID#20 showed that Rat-ID#13
and Rat-ID#20 (F0- *Psen1*^{LF} rats) had no off-target mutations in this site.
WILDTYPE 5'-GCCCGCCCTCCCCATCTCCATCACCTTCGGGCTCATCTTTTACTTCTCCACAGACAACCT-3'
ID#13 5'-GCCCGCCCTCCCCATCTCCATCACCTTCGGGCTCATCTTTTACTTCTCCACAGACAACCT-3'
ID#20 5'-GCCCGCCCTCCCCATCTCCATCACCTTCGGGCTCATCTTTTACTTCTCCACAGACAACCT-3'

Off-target site on chr 19: 45648872 GAAGATGAGACAGAAGGTGAAAG 45648894

Targeting sequence: GAAAATGAGCCCGAAGGTGATGG
A 450bp long products was generated by PCR with the following F and R oligos:
F- CCCTCGAAGTTCCAAACTCAAATTAG; R- GAGTTTAATGGATGCTAGTTTGCCGT
The PCR product was sequenced using primer F. Sequencing results comparing WT rat and Rat-ID#13 and Rat-ID#20 showed that Rat-ID#13
and Rat-ID#20 (F0- *Psen1LF* rats) had no off-target mutations in this site.
WILDTYPE 5'-AGGGACCATCAACGAGCAAGGAAGATGAGACAGAAGGTGAAAGAATCAGACTGTGCTGAG-3'
ID#13 5'-AGGGACCATCAACGAGCAAGGAAGATGAGACAGAAGGTGAAAGAATCAGACTGTGCTGAG-3'
ID#20 5'-AGGGACCATCAACGAGCAAGGAAGATGAGACAGAAGGTGAAAGAATCAGACTGTGCTGAG-3'

Off-target site on chr 5: 171566677 GCAGTTAAGCCCGAAGGTGAGGG 171566699

Targeting sequence: GAAAATGAGCCCGAAGGTGATGG
A 422bp long products was generated by PCR with the following F and R oligos:
F-CAACTTAGCCTTAGGGGCATGACA; R-CATGAGGTCCCAAGTGTCCACC
The PCR product was sequenced using primer R. Sequencing results comparing WT rat and Rat-ID#13 and Rat-ID#20 showed that Rat-ID#13
and Rat-ID#20 (F0- *Psen1*^{LF} rats) had no off-target mutations in this site.
WILDTYPE 5'-AAACAGGTACAAATCTGGCTGCAGTTAAGCCCGAAGGTGAGGGGTGGGCAACCTGCAATA-3'
ID#13 5'-AAACAGGTACAAATCTGGCTGCAGTTAAGCCCGAAGGTGAGGGGTGGGCAACCTGCAATA-3'
ID#20 5'-AAACAGGTACAAATCTGGCTGCAGTTAAGCCCGAAGGTGAGGGGTGGGCAACCTGCAATA-3'

Off-target site on chr 4: 155624036 GAGAGTGACTCCGAAGGTGATAG 155624058

Targeting sequence: GAAAATGAGCCCGAAGGTGATGG
A 353bp long products was generated by PCR with the following F and R oligos:
F-CCCTGTAACTTGTAGGATTTGGTTCCTT; R-TGGTGTGCATGGCTAGTTGTGC
The PCR product was sequenced using primer F. Sequencing results comparing WT rat and Rat-ID#13 and Rat-ID#20 showed that Rat-ID#13
and Rat-ID#20 (F0- *Psen1*^{LF} rats) had no off-target mutations in this site.
WILDTYPE 5'-TTGTTGTGCTTCTCCAAAACGAGAGTGACTCCGAAGGTGATAGGGGGTTCTCAGGAGAAA-3'
ID#13 5'-TTGTTGTGCTTCTCCAAAACGAGAGTGACTCCGAAGGTGATAGGGGGTTCTCAGGAGAAA-3'
ID#20 5'-TTGTTGTGCTTCTCCAAAACGAGAGTGACTCCGAAGGTGATAGGGGGTTCTCAGGAGAAA-3'

Off-target site on chr 11: 75909784 AAATATGTGCCCGAAGGTGGAAG 75909806

Targeting sequence: GAAAATGAGCCCGAAGGTGATGG
A 599bp long products was generated by PCR with the following F and R oligos:
F-GGCGATTGTAATTTTACCGAGTTTTG; R-GCTGTGGAGAAGTGCGGTTTAGAG
The PCR product was sequenced using primer R. Sequencing results comparing WT rat and Rat-ID#13 and Rat-ID#20 showed that Rat-ID#13
and Rat-ID#20 (F0- *Psen1*^{LF} rats) had no off-target mutations in this site.
WILDTYPE 5'-CACACACAATCTTTAAGAAGAAATATGTGCCCGAAGGTGGAAGAATGTTTATTTTGGGGG-3'
ID#13 5'-CACACACAATCTTTAAGAAGAAATATGTGCCCGAAGGTGGAAGAATGTTTATTTTGGGGG-3'
ID#20 5'-CACACACAATCTTTAAGAAGAAATATGTGCCCGAAGGTGGAAGAATGTTTATTTTGGGGG-3'

Figure S1 part1 – Whole Western Blot images used in Figure 2

Antibody | **Chemiluminescence** | **Nitrocellulose** | **Sample order**

Y188

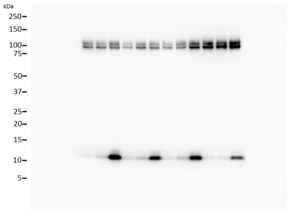
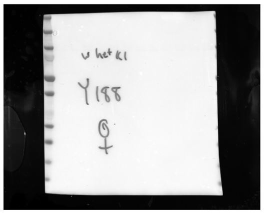

Lane	Sample ID
1.	w/w Female 1
2.	LF/w Female 1
3.	LF/LF Female 1
4.	w/w Female 2
5.	LF/w Female 2
6.	LF/LF Female 2
7.	w/w Female 3
8.	LF/w Female 3
9.	LF/LF Female 3
10.	w/w Female 4
11.	LF/w Female 4
12.	LF/LF Female 4

Y188

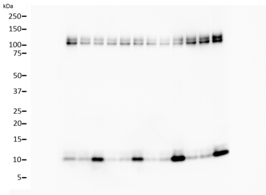
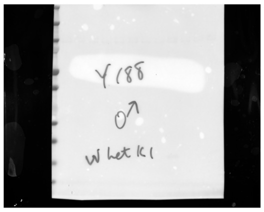

Lane	Sample ID
1.	w/w Male 1
2.	LF/w Male 1
3.	LF/LF Male 1
4.	w/w Male 2
5.	LF/w Male 2
6.	LF/LF Male 2
7.	w/w Male 3
8.	LF/w Male 3
9.	LF/LF Male 3
10.	w/w Male 4
11.	LF/w Male 4
12.	LF/LF Male 4

N-cadherin (2 exposures)

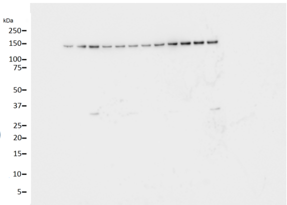
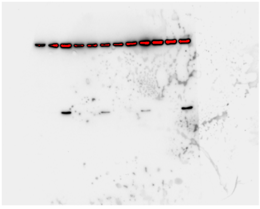
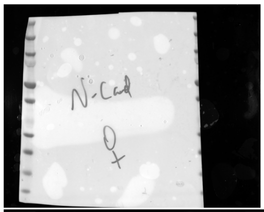

Lane	Sample ID
1.	w/w Female 1
2.	LF/w Female 1
3.	LF/LF Female 1
4.	w/w Female 2
5.	LF/w Female 2
6.	LF/LF Female 2
7.	w/w Female 3
8.	LF/w Female 3
9.	LF/LF Female 3
10.	w/w Female 4
11.	LF/w Female 4
12.	LF/LF Female 4

N-cadherin (2 exposures)

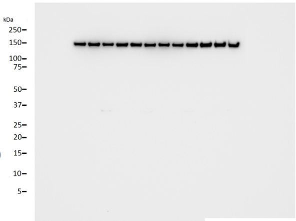
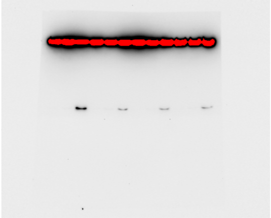
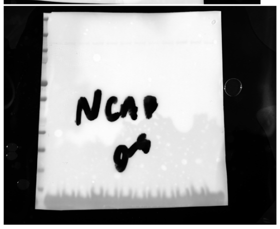

Lane	Sample ID
1.	w/w Male 1
2.	LF/w Male 1
3.	LF/LF Male 1
4.	w/w Male 2
5.	LF/w Male 2
6.	LF/LF Male 2
7.	w/w Male 3
8.	LF/w Male 3
9.	LF/LF Male 3
10.	w/w Male 4
11.	LF/w Male 4
12.	LF/LF Male 4

GAPDH

Note: Male N-cad blot run seperately, but from same samples, requiring its own GAPDH blot

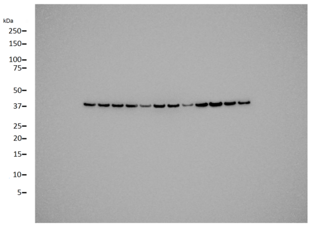
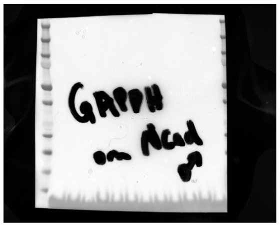

Lane	Sample ID
1.	w/w Male 1
2.	LF/w Male 1
3.	LF/LF Male 1
4.	w/w Male 2
5.	LF/w Male 2
6.	LF/LF Male 2
7.	w/w Male 3
8.	LF/w Male 3
9.	LF/LF Male 3
10.	w/w Male 4
11.	LF/w Male 4
12.	LF/LF Male 4

PS1-CTF

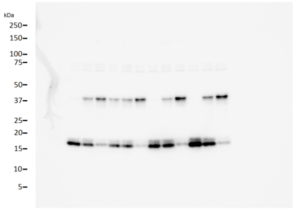
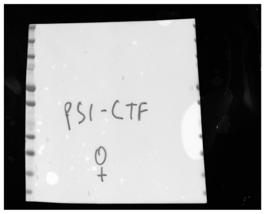

Lane	Sample ID
1.	w/w Female 1
2.	LF/w Female 1
3.	LF/LF Female 1
4.	w/w Female 2
5.	LF/w Female 2
6.	LF/LF Female 2
7.	w/w Female 3
8.	LF/w Female 3
9.	LF/LF Female 3
10.	w/w Female 4
11.	LF/w Female 4
12.	LF/LF Female 4

PS1-CTF

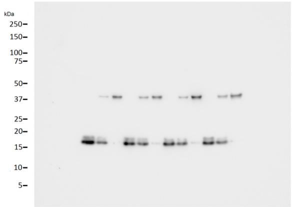
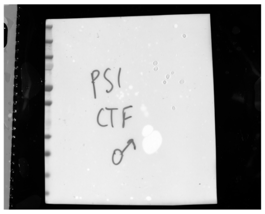

Lane	Sample ID
1.	w/w Male 1
2.	LF/w Male 1
3.	LF/LF Male 1
4.	w/w Male 2
5.	LF/w Male 2
6.	LF/LF Male 2
7.	w/w Male 3
8.	LF/w Male 3
9.	LF/LF Male 3
10.	w/w Male 4
11.	LF/w Male 4
12.	LF/LF Male 4

Figure S1 part2 – Whole Western Blot images used in Figure 2

Antibody | **Chemiluminescence** | **Nitrocellulose** | **Sample order**

PS1-NTF

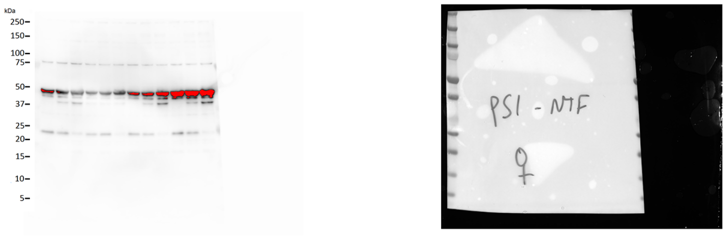

Lane	Sample ID
1.	w/w Female 1
2.	LF/w Female 1
3.	LF/LF Female 1
4.	w/w Female 2
5.	LF/w Female 2
6.	LF/LF Female 2
7.	w/w Female 3
8.	LF/w Female 3
9.	LF/LF Female 3
10.	w/w Female 4
11.	LF/w Female 4
12.	LF/LF Female 4

PS1-NTF

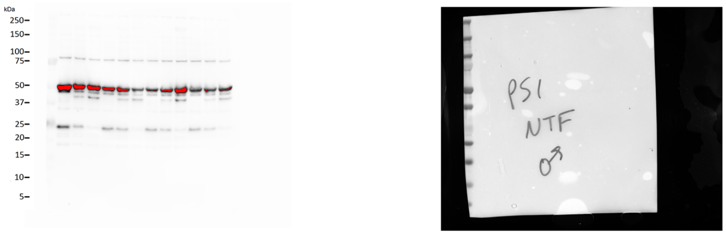

Lane	Sample ID
1.	w/w Male 1
2.	LF/w Male 1
3.	LF/LF Male 1
4.	w/w Male 2
5.	LF/w Male 2
6.	LF/LF Male 2
7.	w/w Male 3
8.	LF/w Male 3
9.	LF/LF Male 3
10.	w/w Male 4
11.	LF/w Male 4
12.	LF/LF Male 4

PS2

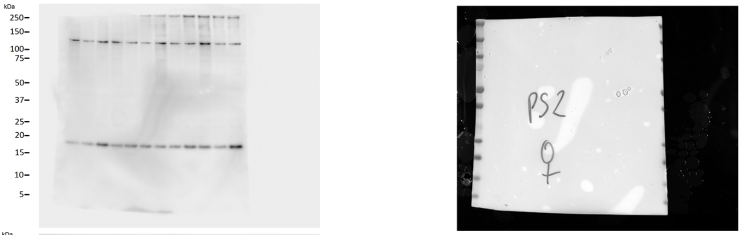

Lane	Sample ID
1.	w/w Female 1
2.	LF/w Female 1
3.	LF/LF Female 1
4.	w/w Female 2
5.	LF/w Female 2
6.	LF/LF Female 2
7.	w/w Female 3
8.	LF/w Female 3
9.	LF/LF Female 3
10.	w/w Female 4
11.	LF/w Female 4
12.	LF/LF Female 4

PS2

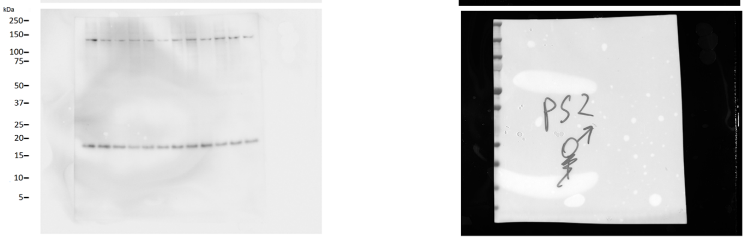

Lane	Sample ID
1.	w/w Male 1
2.	LF/w Male 1
3.	LF/LF Male 1
4.	w/w Male 2
5.	LF/w Male 2
6.	LF/LF Male 2
7.	w/w Male 3
8.	LF/w Male 3
9.	LF/LF Male 3
10.	w/w Male 4
11.	LF/w Male 4
12.	LF/LF Male 4

Nicastrin

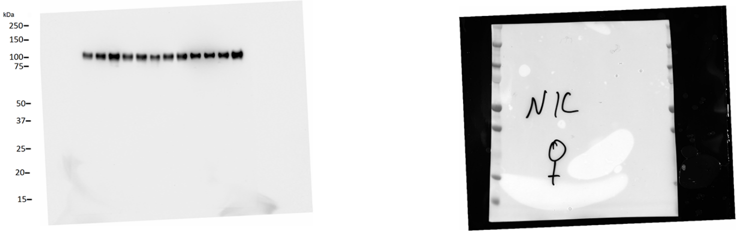

Lane	Sample ID
1.	w/w Female 1
2.	LF/w Female 1
3.	LF/LF Female 1
4.	w/w Female 2
5.	LF/w Female 2
6.	LF/LF Female 2
7.	w/w Female 3
8.	LF/w Female 3
9.	LF/LF Female 3
10.	w/w Female 4
11.	LF/w Female 4
12.	LF/LF Female 4

Nicastrin

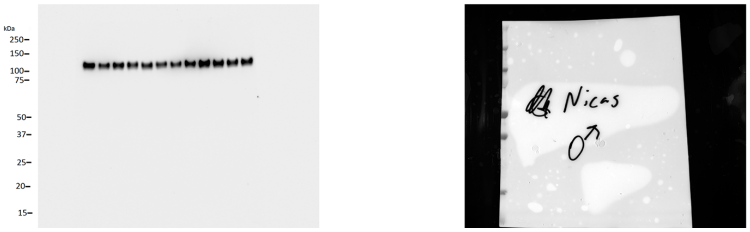

Lane	Sample ID
1.	w/w Male 1
2.	LF/w Male 1
3.	LF/LF Male 1
4.	w/w Male 2
5.	LF/w Male 2
6.	LF/LF Male 2
7.	w/w Male 3
8.	LF/w Male 3
9.	LF/LF Male 3
10.	w/w Male 4
11.	LF/w Male 4
12.	LF/LF Male 4

Figure S1 part3– Whole Western Blot images used in Figure 2

Antibody	Chemiluminescence	Nitrocellulose	Sample order

GAPDH

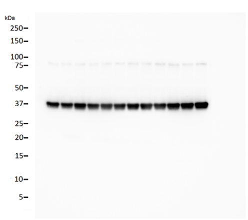

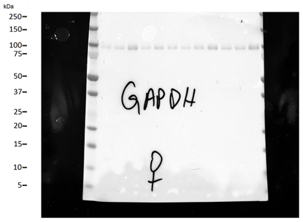

Lane	Sample ID
1.	w/w Female 1
2.	LF/w Female 1
3.	LF/LF Female 1
4.	w/w Female 2
5.	LF/w Female 2
6.	LF/LF Female 2
7.	w/w Female 3
8.	LF/w Female 3
9.	LF/LF Female 3
10.	w/w Female 4
11.	LF/w Female 4
12.	LF/LF Female 4

GAPDH

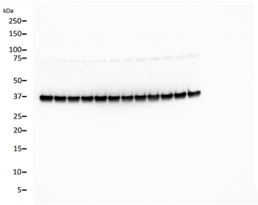

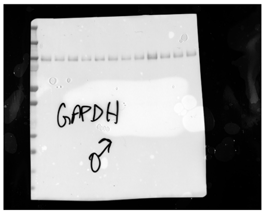

Lane	Sample ID
1.	w/w Male 1
2.	LF/w Male 1
3.	LF/LF Male 1
4.	w/w Male 2
5.	LF/w Male 2
6.	LF/LF Male 2
7.	w/w Male 3
8.	LF/w Male 3
9.	LF/LF Male 3
10.	w/w Male 4
11.	LF/w Male 4
12.	LF/LF Male 4

6E10

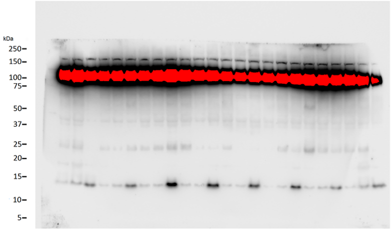

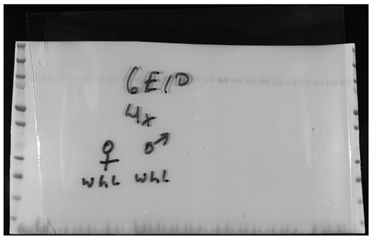

Lane	Sample ID	Lane	Sample ID
1.	w/w Female 1	13.	w/w Female 3
2.	LF/w Female 1	14.	LF/w Female 3
3.	LF/LF Female 1	15.	LF/LF Female 3
4.	w/w Male 1	16.	w/w Male 3
5.	LF/w Male 1	17.	LF/w Male 3
6.	LF/LF Male 1	18.	LF/LF Male 3
7.	w/w Female 2	19.	w/w Female 4
8.	LF/w Female 2	20.	LF/w Female 4
9.	LF/LF Female 2	21.	LF/LF Female 4
10.	w/w Male 2	22.	w/w Male 4
11.	LF/w Male 2	23.	LF/w Male 4
12.	LF/LF Male 2	24.	LF/LF Male 4

GAPDH

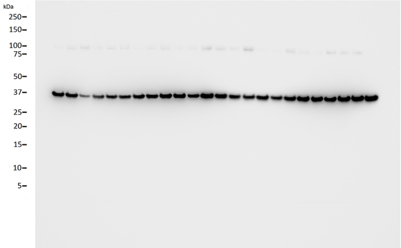

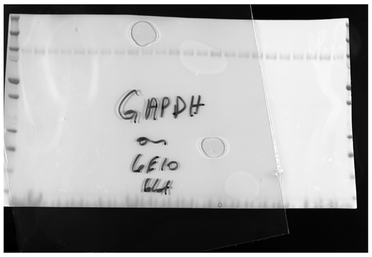

Lane	Sample ID	Lane	Sample ID
1.	w/w Female 1	13.	w/w Female 3
2.	LF/w Female 1	14.	LF/w Female 3
3.	LF/LF Female 1	15.	LF/LF Female 3
4.	w/w Male 1	16.	w/w Male 3
5.	LF/w Male 1	17.	LF/w Male 3
6.	LF/LF Male 1	18.	LF/LF Male 3
7.	w/w Female 2	19.	w/w Female 4
8.	LF/w Female 2	20.	LF/w Female 4
9.	LF/LF Female 2	21.	LF/LF Female 4
10.	w/w Male 2	22.	w/w Male 4
11.	LF/w Male 2	23.	LF/w Male 4
12.	LF/LF Male 2	24.	LF/LF Male 4

Note: 6E10 blot was run with different loading preparations and in a different order, but used the same animal material as all other blots. This required this blot to be reprobed with GAPDH.

Pen2

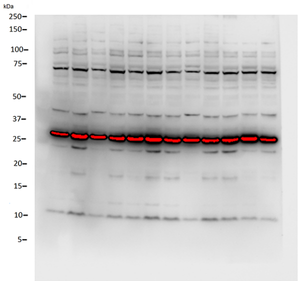

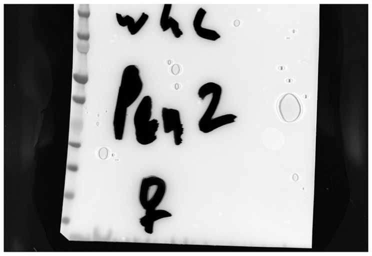

Lane	Sample ID
1.	w/w Female 1
2.	LF/w Female 1
3.	LF/LF Female 1
4.	w/w Female 2
5.	LF/w Female 2
6.	LF/LF Female 2
7.	w/w Female 3
8.	LF/w Female 3
9.	LF/LF Female 3
10.	w/w Female 4
11.	LF/w Female 4
12.	LF/LF Female 4

Pen2

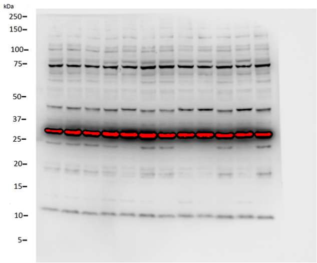

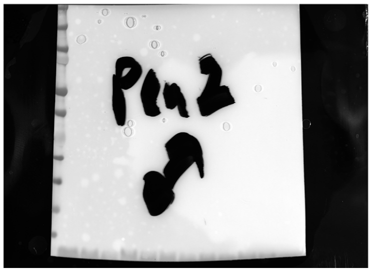

Lane	Sample ID
1.	w/w Male 1
2.	LF/w Male 1
3.	LF/LF Male 1
4.	w/w Male 2
5.	LF/w Male 2
6.	LF/LF Male 2
7.	w/w Male 3
8.	LF/w Male 3
9.	LF/LF Male 3
10.	w/w Male 4
11.	LF/w Male 4
12.	LF/LF Male 4

Figure S2 – Validation of Aβ43, IBL Human Amyloidβ (1-43) (FL) Assay Kit (27710) by us using a rat *App*$^{\delta 7/\delta 7}$ hypomorph control lysates. This Kit can specifically detect human Aβ43 in brain lysates from *App*$^{h/h}$ but not from *App*$^{\delta 7/\delta 7}$ hypomorph controls. Unpaired t test, two-tailed, P<0.0001.

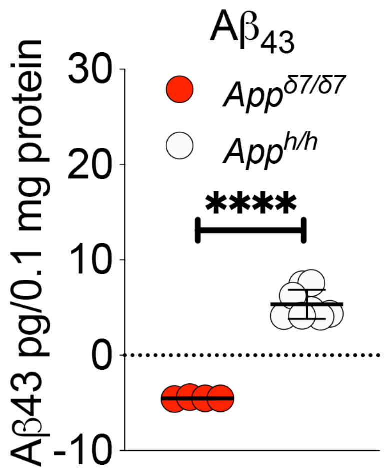

Supplement: Supporting Information [file supp_RA120.012542_157650_2_supp_505283_q8d99t.pdf]
